# Virtual reality simulation—the future of orthopaedic training? A systematic review and narrative analysis

**DOI:** 10.1186/s41077-020-00153-x

**Published:** 2021-01-13

**Authors:** Elinor Clarke

**Affiliations:** University of Warwick Medical School, Coventry, UK

**Keywords:** Virtual reality, Orthopaedics, Education, Training, Surgery, Simulation

## Abstract

**Background:**

Virtual reality (VR) simulation provides users with an immersive, 3D experience that can be used to allow surgical trainees to practice skills and operations in a safe yet realistic environment. The field of orthopaedics is yet to include VR in core teaching, despite its advantages as a teaching aid, particularly against current simulation tools. This study aims to conduct a systematic review to investigate the efficacy of VR in orthopaedic training, against current methods.

**Methods:**

A systemic review of databases Medline, Embase and the Cochrane Library for randomized controlled trials focusing on VR training against conventional training in orthopaedic surgery was performed. Data synthesis was performed through narrative analysis due to the heterogeneous nature of the data.

**Results:**

A total of 16 studies from 140 titles were identified, across 6 specialty areas. Four hundred and thirty-one participants were included. Control groups included VR, cadaver and benchtop simulators. Forty-seven outcomes were measured, focusing on skill and proficiency assessment. No outcomes focused on patient safety. Although significance between intervention and control was not always achieved, most studies found that the intervention outperformed the control.

**Conclusion:**

VR provides a modern and immersive teaching tool that can develop skills and give confidence to trainees. This study demonstrates the potential for VR simulation as a training aid in orthopaedics and encourages its use alongside conventional teaching methods. However, long-term analysis of the results of VR training on surgical trainees has yet to be conducted. To provide conclusive justification for its inclusion in surgical training, this study recommends that future research follows trainees using VR into the operating room, to determine that VR teaches skills that are transferable onto actual surgeries, subsequently leading to better patient outcomes.

## Background

Simulation is an essential component in medical education, in that it allows trainees to develop the skills required in an environment that does not compromise patient safety. The surgical field of orthopaedics has a well-established history in the area of simulation, and these tasks largely involve low-fidelity models, or the use of wet or dry labs for anatomical learning [1]. However, these models may not as accurately represent the surgical environment or require sufficient access to resources that may not always be freely available and, in some cases, can only be used once, i.e. human tissue. Teaching within the operating room itself has served as a solution for many years, but is problematic, due to the ethical and safety concerns that arise in introducing inexperienced trainees to complicated procedures in high pressure environments [2].

Virtual reality (VR)—simulation technology that allows users to become immersed in and interact with a 3D, computer-generated environment in real time—has been discussed in the context of medical and surgical education for decades [3]. The significant appeal that VR simulation provides is that it allows operations—in full, or in part—to be practised, and the outcome viewed, before the patient enters the surgery. Because of this, surgical approaches can be adjusted and rehearsed, with clear advantages for patients and healthcare providers. Beyond the rehearsal and refinement of procedures, VR lends itself to being an excellent teaching tool, providing trainees of all level access to a range of techniques that accurately replicate real-life environments, without risk to the patient or even a necessary need for supervision [4].

Despite the advantages that VR training provides, it is not commonly used as part of core surgical curriculum. VR technology may be particularly useful in orthopaedics, due to the specific mechanical nature of techniques that trainees are required to learn, where prior practise and repetition of skills is important in developing sufficient competency. Currently, VR simulation in orthopaedic education is effectively non-existent [1]. VR, therefore, may provide a long term and sustainable alternative that presents a modern and immersive solution to building surgical confidence and competency.

Research into the use of VR in orthopaedics specifically has appeared for over 2 decades. In 1998, Blackwell et al. [5] hypothesised potential uses of ‘augmented reality technology’ to provide simulated views of joints, heightened visualisation of anatomical structures and decreased surgical complications by minimising damage to surrounding tissue. More recently, as technologies develop and become more mainstream, validity studies determined the positive correlation between surgical experience and VR performance [6–8], and a 2015 systematic review by Aim et al. [9] concluded that although VR was promising, data was limited—indeed, only 9 studies were included in analysis. Since the publication of Aim et al.’s review, there has been an increase in trials examining VR in orthopaedic training, particularly designed as RCTs. And yet, VR appears to be still a technology ‘of the future’, and as is demonstrated in recent publications by the British Orthopaedic Association in their training guidelines [1], there is little to no indication of a hurry to incorporate VR simulation into curriculum, despite the long-standing anticipation of previous researchers.

With the continual publishing of research exploring the effectiveness of VR simulation against current practices, it is important for new systematic reviews such as this one to provide analysis and commentary. As such, it is the aim that by providing continual trend analysis and further developing evidence of both the successes and limitations of VR simulation, this will increase its recognition as a valuable teaching tool. There remains a place within the research for the synthesis that this study aims to provide, to give further up-to-date evidence that informs and pushes to develop current practise.

This study aims to conduct a systematic review of relevant literature and analyse the efficacy of VR simulation in orthopaedic surgical training, with a focus on outcomes in comparison to current standard training methods. The question this paper will be asking is does training in VR lead to a greater positive effect on outcomes that reflects real surgical competence, compared to standard training currently used in the orthopaedic curriculum, for surgical trainees of all levels.

## Methods

### Search methods for identification of studies

Searches for eligible studies were conducted through online databases, including Medline, the Cochrane Library and Embase.

Search terms included virtual reality, VR, computer simulation, orthop*, arthrop* and surgery, and were appropriately altered and expanded upon for each database (Table 1). Additionally, the reference lists of identified studies were screened, as well as previous relevant systematic reviews [9, 10]. Titles, abstracts and subsequently full papers were screened for relevancy and data extraction.

**Table 1:** Databases and according search strategy

Database	Search strategy	Items found
Medline	1. ((((virtual reality[MeSH Terms]) OR virtual realt*[Title/Abstract]) OR computer simulation[MeSH Terms]) OR virtual simulat*[Title/Abstract]) OR vr[Title/Abstract] 2. (((((orthopedic[MeSH Terms]) OR arthroplasty[MeSH Terms]) OR arthroplasty, replacement, hip[MeSH Terms]) OR arthroplasty, replacement, knee[MeSH Terms]) OR shoulder[MeSH Terms]) OR spine[MeSH Terms] 3. Surgery 4. (((((((activities, training[MeSH Terms]) OR academic training[MeSH Terms]) OR training) OR activities, educational[MeSH Terms]) OR education) OR trainees) OR task performances, analysis[MeSH Terms]) OR clinical competence[MeSH Terms] 5. #1 AND #2 AND #3 AND #4	167
Cochrane Library	1. MeSH [Virtual Reality] explode all trees 2. MeSH [Computer Simulation] this term only 3. MeSH [Orthopedics] explode all trees 4. MeSH [Arthropathy, Neurogenic] in all MeSH products 5. VR OR Computer Instruction 6. Shoulder OR knee OR hip OR spine OR elbow 7. Surgery 8. #1 OR #2 OR #5 9. #3 OR #4 OR #6 10. #8 AND #9 AND #7	152
Embase	1. Virtual reality 2. Virtual reality simulator 3. Computer simulation 4. VR 5. Ortho* 6. Arthro* 7. Knee 8. Shoulder 9. Elbow 10. Spine 11. Ankle 12. Training or Surgical training or Simulation training 13. Trainees or student or resident 14. Task performance 15. Virtual reality OR virtual reality simulator OR computer simulation OR VR 16. Orthop* OR arthro* OR knee OR shoulder OR elbow OR spine OR ankle 17. Training or surgical training or simulation training OR trainees or student OR task performance 18. #15 AND #16 AND #17	300

### Criteria for eligibility

The research question being asked is does training in VR lead to a greater positive effect on outcomes that reflect real surgical competence, compared to standard training? The PICO criteria for study inclusion are as shown in Table 2.

**Table 2:** ‘Population Intervention Comparison Outcomes’ (PICO) criteria for eligibility

**Population**	Medical trainees ranging from medical students to consultant level.
**Intervention**	VR training in orthopaedic surgery. Not restricted to specific surgical procedures or type of joint.
**Comparison**	No training/standard training/other simulation types.
**Outcomes**	Surgically relevant outcomes, including time to complete part or all of a procedure, damage to tissue and surgical skill checklists.

### Types of studies

Randomised, controlled trials (RCTs) were included. Alternative study designs including observational studies were not eligible.

Country of origin was not a limiting factor. Only English language studies were included.

### Data extraction and synthesis

Each study eligible for data extraction was tested against CASP criteria [11] for critical appraisal and Robvis [12] for risk of bias before continuing with data synthesis.

Due to the heterogeneous data and methodology in the eligible articles, statistical analysis was not possible, and a narrative analysis was performed. Data extracted included specialty of focus (i.e. knee, hip, shoulder), participant number and level of training, VR simulator model, the simulated task and assessment, outcome measures and main conclusions drawn through study results.

## Results

**Fig. 1:**
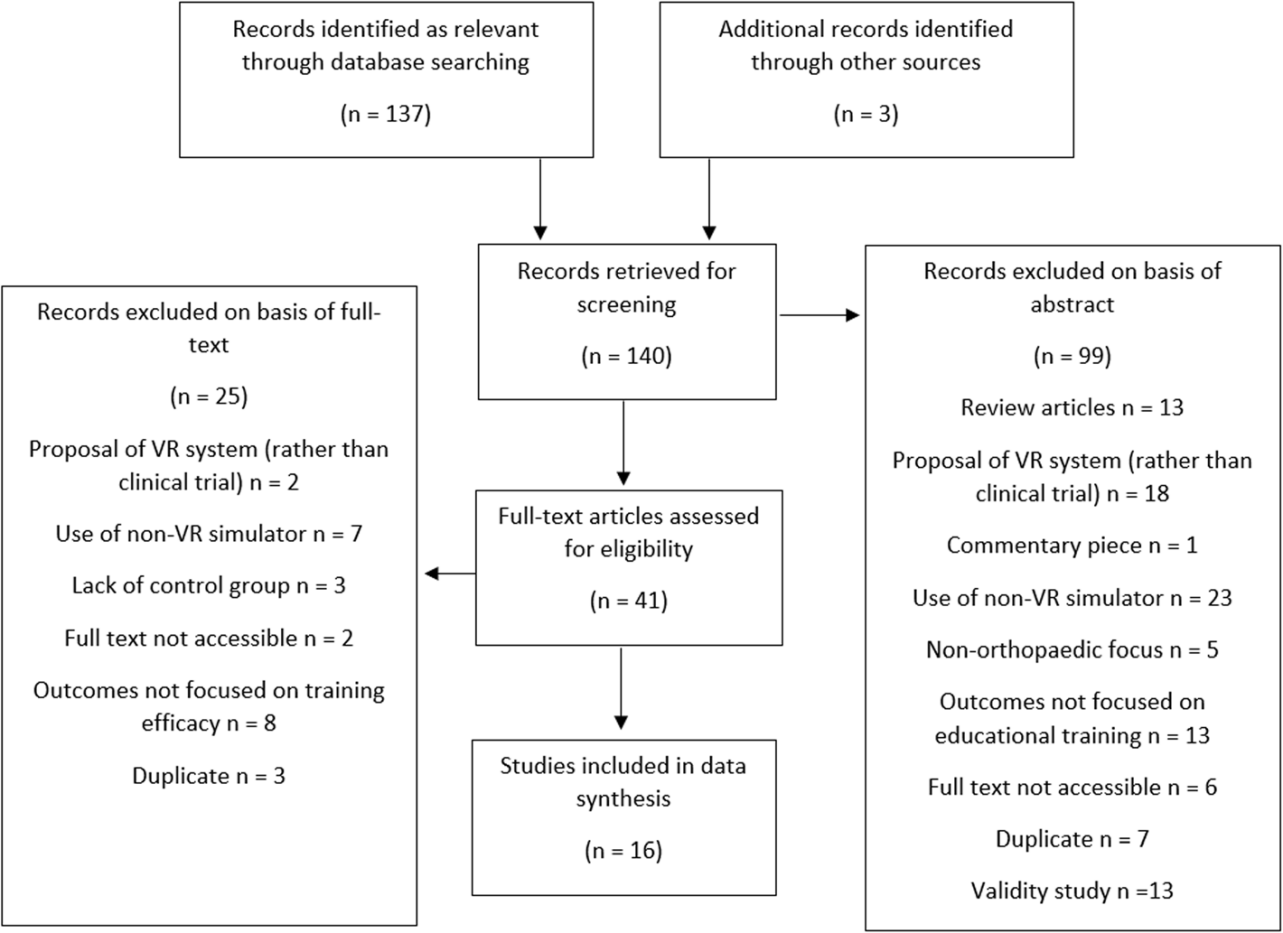
PRISMA flowchart illustrating the refinement of potential studies for review. After 140 initial potential studies, 16 are taken onto thematic analysis

A total of 140 titles were identified as being potentially relevant and were narrowed down during abstract and full-text analysis (Fig. 1). Studies were excluded for a number of reasons, including a non-orthopaedic focus and using a simulator that would not be classed as VR. The total number of studies taken onto thematical analysis was 16.

### Study characteristics

Nine out of 16 articles focused on arthroscopy. Of this 9, 4 focused on shoulder arthroscopy [13–17] and 5 on knee arthroscopy [16, 18–21]. Rebolledo et al. [16] were the only researchers to focus on 2 areas of simulation, with both knee and shoulder arthroscopy skills included. The second most common area of focus was spinal pedicle screw placement (3 out of 16) [22–24]. Other procedures included tibial shaft fracture fixation [25], pre-surgery fracture carving [26], dynamic hip screw placement [27] and hip arthroplasty [28].

A total of 13 different VR simulators were used. Most commonly used was ArthroSim, included in 3 articles, all of which for knee arthroscopy [19–21]. ArthroVR was used in 2 articles [17, 18], as was insightArthro [13, 16]. The remainder of the simulators were used in only 1 article each—Osso VR [25], Immersive Touch [22], Procedius [14], IVRSS-PSP [24], ORamaVR [28], VSTS [23], PrecisionOS [15], Virtual-Fracture-Carving-Simulator [26] and TraumaVision [27].

Four hundred and thirty-one participants were included in analysis. Participants were ranged in experience level from medical students with no surgical experience to surgical ‘experts’, the definition of which differed across papers. Participant characteristics can be found in Table 3 and numbers of participants in each study in Table 4.

**Table 3:** Participant characteristics

Participant characteristics	Reported totals
Novices (no previous experience; medical students or junior doctors)	253 (reported in 16 articles)
Intermediates (limited experience, not primary surgeon; surgical trainees)	141 (reported in 16 articles)
Experts (extensive experience, primary surgeons; high level trainees, consultants)	37 (reported in 16 articles)
Female:Male	62:155 (reported in 6 articles)

**Table 4:** Study descriptions

	Population	Methodology				
Study	No. of participants	VR-simulated task simulator	Control Task	Assessment	Outcome Measures	Results
Andersen et al. [13]	21 (14 novices, 7 experts)	Visualise, palpating spheres on anatomical landmarks in the shoulder	No additional training	Visualise, palpating spheres on anatomical landmarks in VR	Time to complete exercise, number of collisions, maximum depth of collision, paths travelled by camera and probe	SS reduction in time (*p* = 0.03), path distance (*p* = 0.02) and depth of collisions (*p* = 0.02) for VR group. Number of collisions not SS in difference (*p* = 0.07).
Banaszek et al. [18]	40 (all novices)	Perform diagnostic knee arthroscopy and probing examination	Control 1: perform simulated task on benchtop simulator Control 2: no additional training	Perform diagnostic arthroscopy on both simulators and cadaver, perform medial meniscectomy on cadaver	GRS scores, procedure-specific checklist, time per task, motion analysis	Both simulator groups showed improvement compared to control in all outcomes. VR group performed SS better than benchtop in lab and on cadaver (*p* = 0.02).
Blumstein et al. [25]	17 (all novices)	Perform tibial shaft fracture IM nailing	Read printed instructions on surgical technique for procedure	Perform tibial shaft fracture IM nailing on benchtop model	GRS and procedure-specific checklist	SS higher GRS (*p* = 0.001) and increase in correctly completed steps (*p* = 0.008) in VR group.
Cannon et al. [19]	48 (all intermediates)	Visualise, probe anatomical structures in the knee (must achieve level of proficiency to progress)	No additional training	Perform diagnostic arthroscopy on live patient, within 25 min	GRS, procedure-specific checklist (visualisation scale and probing scale)	VR group had SS higher scores in procedure-specific checklist (*p* = 0.031), but not GRS (*p* = 0.061). Visualisation score did not have SS difference (*p* = 0.34). Control group was faster but performed less correct steps.
Cychosz et al. [20]	43 (all novices)	Complete FAST modules on tracking, periscoping, palpation and collecting stars. Perform knee arthroscopy	No additional training	Perform diagnostic knee arthroscopy in VR	Camera path length, cartilage damage, time to complete	VR group had SS higher overall scores (*p* = 0.046) and shorter path length (0.0274). Time and damage not SS in difference (*p* = 0.3, *p* = 0.4). VR group showed greater level of improvement pre- and post-test.
Gasco et al. [22]	26 (all novices)	Place 2 pedicle screws	Didactic lecture on surgical technique for procedure	Place 2 pedicle screws in benchtop model	Screw placement, choice of screw, pedicle breaches	SS less errors in all outcomes for VR group (more than 50% reduction in placement error (*p* < 0.001))
Henn et al. [14]	17 (all novices)	Touch 11 targets in the shoulder	No additional training	Probe-specific points within shoulder on cadaver	GOALS score (time to complete, dexterity, depth perception, efficiency, respect for tissue)	SS reduction in time for VR group (*p* < 0.05), with SS improvement from baseline (*p* < 0.05). Improvement in GOALS score was greater than control, but not SS (*p* = 0.98)
Hooper et al. [28]	14 (all novices)	Perform 2 simulated THAs	No additional training	Perform THA on cadaver	THA score, GRS	VR group showed greater improvement from baseline in all outcomes; however, this was not SS (*p* = 0.078). Only technical performance was SS (*p* = 0.009)
Hou et al. [23]	10 (all novices)	Perform pedicle screw placement	Didactic lecture and video on surgical technique for procedure	Perform cervical pedicle screw placement on cadaver	Screw placement	SS higher ‘acceptable’ screw placement in VR group (100% vs 50% in control group, *p* = <0.05). SS higher ‘ideal’ screw placements for VR group (*p* = <0.05)
Lohre et al. [15]	26 (19 intermediates, 7 experts)	Complete module outlining key steps in glenoid exposure procedure	Read technical article outlining steps of procedure	Perform glenoid exposure on cadaver	Time to complete, OSATS, completion of procedure-specific checklist	SS reduction in time for VR group (*p* = 0.04). Improvement in OSATS score, however only SS improvement over control in instrument handling (*p* = 0.03)
Middleton et al. [21]	17 (all novices)	Visualisation, probing of anatomical structures within the knee	Visualisation, probing of anatomical structures within the knee on benchtop simulator	All groups perform visualisation and probing of anatomical structures within the knee on both benchtop and VR simulators	Motion analysis (total time taken and number of hand movements), GRS	Both groups improved from baseline (*p* = <0.05). Control group showed SS improvement on VR test (*p* = <0.05), but VR group did not show SS improvement on benchtop test (*p*= > 0.05). VR group did not consistently outperform control group on VR test.
Pahuta et al. [26]	48 (all novices)	Drawing of both column hemipelvis fracture lines	Control 1: draw fracture lines on benchtop model Control 2: read article on fracture carving, view 3D CT images	Drawing of both column hemipelvis fracture lines on surgically arranged benchtop hemipelvis, in 5 min	Accuracy of drawn fracture lines against known anatomical features of both-column fractures	VR group performed SS better than both control groups (*p* = 0.0001, *p* = 0.0026); lines were more accurate and had correct spatial relationships. No SS difference between control groups.
Rebolledo et al. [16]	14 (all novices)	Probing of spheres on anatomical landmarks in the knee and shoulder	2 h of didactic lectures on surgical technique	Perform standard diagnostic arthroscopy on knee and shoulder cadaver model	Time to complete, generated injury grading index (dexterity, collisions, injury to tissue)	SS reduction in time (*p* = 0.02) and injury grading index (*p* = 0.01) for VR group in shoulder exercises. VR group performed better than control in knee exercises, but differences were not SS (*p* = 0.09, *p* = 0.08)
Sugand et al. [27]	52 (all intermediates)	Perform fixation of intertrochanteric fracture, 5x a week for 2 weeks	Perform fixation of intertrochanteric fracture, 1x a week for 2 weeks	Perform fixation of intertrochanteric fracture in VR	Time to complete, total fluoroscopy time, number of attempts to place guidewire, GRS	VR group performed better than control with SS in all outcomes (*p* = <0.001). VR also showed greater improvement from baseline than control.
Waterman et al. [17]	22 (all intermediates)	Location of spheres in anatomical locations in shoulder, palpation of spheres	No additional training	Perform shoulder arthroscopy on live patient	Time to complete, camera distance, probe distance, ASSET	VR group had SS improvement from baseline (*p* = 0.01). VR group was SS faster than control (*p* = 0.01). ASSET score and camera distance were better the VR group, however without SS (*p* = 0.061, *p* = 0.070).
Xin et al. [24]	16 (all intermediates)	Perform pedicle screw placement	Watch demonstration of correct nail placement and technique on 3D-printed model.	Placement of 6 pedicle screws T11-L4 on cadaver	Time for each screw, position of screw	SS higher ‘acceptable’ screw placement in VR group (100% vs 79.2% in control group, *p* = <0.05). SS higher ‘ideal’ screw placements for VR group (*p* = <0.05). SS reduction in time for VR group (*p* = <0.05).

*VR* virtual reality, *SS* statistically significant, *GRS* Global Rating Scale, *IM* intramedullary, *FAST* Fundamentals of Arthroscopic Surgery Training, *GOALS* Global Operative Assessment of Laparoscopic Skills, *THA* total hip arthroscopy, *OSATS* Objective Structured Assessment of Technical Skills, *ASSET* Arthroscopic Surgery Skill Evaluation Tool

The simulated task participants completed varied across articles, as well as methods of assessment (Table 4).

Studies focusing on arthroscopies used simulated tasks in the intervention group that were broadly similar; visualisation and probing of prompted anatomical landmarks or the location of virtual shapes within the joint space. The 3 studies focusing on spinal pedicle placement and the 4 studies that had unique focuses followed simulated tasks that directly embodied the procedure they were replicating.

The choice of task for the control group also varied. Seven studies chose to have their control group receive no additional learning to complete before assessment [13, 14, 17–20, 28], 6 had their control groups receive didactic lectures or demonstrations, or read instruction manuals on the relevant surgical technique [15, 16, 22, 23, 25, 26], 3 used SawBones—a benchtop simulator—as their control [18, 21, 26], and the remaining 2 had unique control group tasks, including using the same VR simulator as the intervention group for a much shorter amount of time [24, 27].

The locations for assessment of participants can be found in Table 5. Only 2 studies performed the assessment on live patients in the operating room—both shoulder arthroscopies [17, 19]. Most commonly used was cadaver [14–16, 18, 23, 24, 28], followed by VR [13, 18, 20, 21, 27] and benchtop [18, 21, 22, 25, 26].

**Table 5:** Outcome measures and methods of assessment

	No. of studies
**Outcomes**	
Time to complete task	10
GRS	6
Procedure-specific checklist	5
Path length (camera)	3
Screw placement	3
Path length (probe)	2
Tissue damage	2
Motion analysis	2
ASSET	1
OSATS	1
GOALS	1
Number of collisions	1
Total fluoroscopy time	1
Number of guidewire attempts	1
Accuracy of drawn fracture lines	1
Screw choice	1
Injury grading index	1
**Method of assessment**	
Cadaver	7
VR Simulator	5
Benchtop simulator (SawBones)	5
Operating room (live patient)	2

### Outcome measures

Forty-seven outcomes were measured across the 16 articles, which covered 17 topics (Table 5). Time to complete the simulated task was measured in the greatest number of articles (10) [13–18, 20, 21, 24, 27], and several established surgical skill checklists (Global Rating Scale (GRS) [18, 19, 21, 25, 27, 28], Global Operative Assessment of Laparoscopic Skills (GOALS) [14], Objective Structured Assessment of Technical Skills (OSATS) [15], Arthroscopic Surgery Skill Evaluation Tool (ASSET) [17]) were used, alongside procedure-specific checklists that were designed for the study by the researchers [15, 18, 19, 25, 28]. Of the 17 outcome areas, only 6 were reported in more than 2 studies. All outcomes were focused on the skill and proficiency of participants during assessment, as a representation of the effectiveness of the intervention simulator. Notably, in the articles that assessed participants in the operating room, there were no outcomes focused on patient safety, procedure outcome or complications.

### Study results

Both pre-test and post-test assessment were completed in 8 studies [13, 14, 17, 18, 20, 21, 27, 28], establishing a participant baseline.

In all 8 studies, the intervention group demonstrated an improvement from baseline, and all studies bar 2 [13, 28] noted a statistically significant difference in at least one outcome. All studies found the improvement to be greater than that of the control group. Statistical significance between intervention and control was not always achieved, though most studies found that the intervention outperformed the control.

The notable exception to this is Middleton et al. who used a benchtop simulator as their control and tested both groups on both simulators. They identified that the VR group did not outperform the control on the benchtop simulator, or on the VR simulator, and suggested that benchtop simulators may provide more generic, transferable motor skills.

The remaining 8 studies [15, 16, 19, 22–26] were compared between groups after training and did not record a participant baseline. All 8 studies found that the VR group outperformed the control, and 6 achieved statistical significance for the VR group in all outcomes measured [22–26]. The only outcome in which the control achieved ‘better’ results was for time to complete the task [19]; however, the control group also performed less correct steps in the procedure than the VR group.

### Risk of bias assessment

**Fig. 2:**
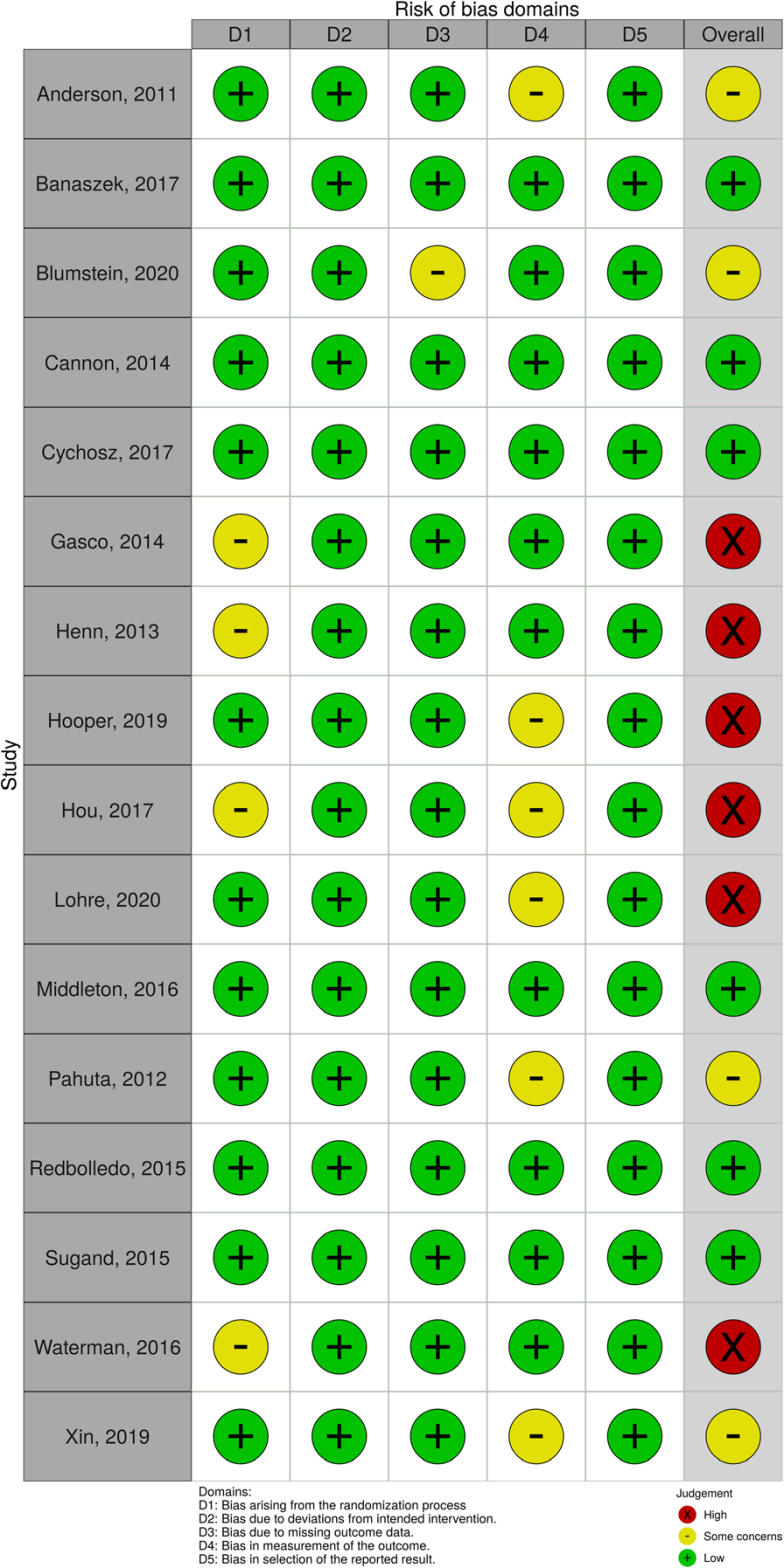
Risk of bias traffic light plot

Risk of bias assessments were completed for each article (Fig. 2) using Robvis [12]. While the data was generally assessed to be at a low risk of bias, there were a few exceptions. Four articles did not note what randomisation technique they used to divide participants between groups [14, 17, 22, 23]. One article reported a loss of participants during the trial, potentially leading to missing data [25], 2 used multiple assessors without incorporating a method of reducing subsequent assessor bias [15, 28], which Hooper et al. acknowledged lead to disparities in their results, and 4 [13, 17, 24, 26] made no mention of blinding assessors.

### CASP analysis

Studies were critically appraised against a CASP [11] RCT checklist. Overall, the studies were found to be of an acceptable quality. However, there were, again, some concerns over randomisation [14, 17, 22, 23]. Full blinding is difficult to achieve in educational studies, as participants usually know what group they are in; therefore, only assessors can be made blind; this was achieved in 10 studies [14–17, 19, 21, 22, 25, 28]. Three studies used assessment data generated from the VR simulator itself, which provided a completely objective measurement [20, 21, 27]. Establishing similarity between groups at the start of the trial was attempted by 12 studies [13, 15–21, 24–27] and was performed particularly well by Cannon et al. [19] and Pahuta et al. [26] who undertook hand-eye-coordination testing on participants alongside skill checks before randomisation.

It was deemed that the results of all the studies will help locally, in that they produced contextual results that are clinically relevant, with clear benefits to the population.

## Discussion

Virtual reality technology is increasingly being integrated into teaching in medicine, and beyond. However, VR simulation is rarely incorporated into orthopaedic training.

This study aimed to analyse the effectiveness of VR training in orthopaedics. Through database searching, a total of 16 RCTs were identified. These studies used a range of controls, including low-fidelity benchtop models and lecture-style teaching.

Of the 16 studies, 15 determined that trainees using VR simulations perform better than those using standard training methods in outcomes including validated surgical skill checklists. A total of 47 outcomes were measured across the studies, and 29 of these achieved statistical significance for VR over the associated control. On the surface, therefore, this result could lead to the conclusion that training in VR does lead to a greater positive effect on outcomes than standard training currently used in the orthopaedic curriculum. However, there are still several concerns related to the effectiveness of VR despite the apparent positive outcomes seen by studies examined in this review.

In previous reviews analysing this subject [9], articles exclusively focused on arthroscopy. Since then, trials have expanded across the orthopaedic specialty, and this study identified articles across 5 areas of orthopaedics. This expansion is due largely to the ongoing development of new simulators and allows us to view the effectiveness of VR teaching in a wider range of contexts. However, this also contributed to the heterogenicity of data, making fair comparisons across studies more difficult—of the 17 different outcome areas identified, only one was present in more than half of the studies. This heterogenicity largely stemming from a lack of universally accepted methodology and objective assessment has been described as a ‘major concern waiting to be addressed’ [29] for VR use in orthopaedic teaching and is still a fundamental blocking point for VR, limiting validity in measures of proficiency across simulators and surgery types.

Additionally, there is evidence of limited efficacy of VR as a learning tool when applying teaching models to the data. According to Kirkpatrick’s Four Levels [30], evaluating the efficacy of teaching methods involves the analysis of behaviour changes and the long-term impact on outcomes that the teaching provides.

The third level—adaptation of behaviour as a result of teaching—is touched upon by Waterman et al. [17] and Cannon et al. [19] in their testing of participants in the operating room, on real patients. These provide the most complete demonstration of VR’s ability to provide actual, sufficient training that is transferable to the real-life scenario it is trying to emulate.

Both Cannon et al. and Waterman et al. noted that the group training with VR performed better than control when measured with a surgical skill checklist. This improvement in skills has similarly been recorded by research in other surgical fields; Thomsen et al. [31] noted a significant increase in participants score in the OR after VR training in cataract surgeons, while Seymour et al. [32] found VR-trained surgeons to be faster, safer and less likely to make errors in cholecystectomies than non-VR-trained surgeons. However, none of these studies compared VR to another form of simulation as their control, so while it can be said that VR helps participants to perform surgery with more efficacy than someone who did not have training, it cannot be concluded that VR helps participants to perform better in the OR than another form of simulation more widely used. Notably, Waterman et al. did not find a significant post-training improvement in a surgical safety checklist for the VR group, which may suggest that VR training alone does not engage students to actively maintain a high level of patient safety within the surgery.

The practise of using VR as an isolated skills-acquisition tool—as demonstrated by all of the studies included in this analysis—is unlikely to fully prepare trainees for the entire responsibilities expected of a surgeon during a procedure, including essential pre-, mid- and post-op safety checks. The ‘unique selling point’ of VR, and what may make it particularly attractive in surgical training, is its attempts at life-like replications of individual procedures. However, it could be argued that in order to fully achieve this goal of developing an entirely realistic surgical experience, a more holistic view of training within the clinical environment must be taken, and that patient safety should not be viewed as lesser importance than skill development. This ‘whole-scenario’ approach has been seen to be advantageous for users training in acute medicine, where there is an increasingly common usage of simulation suites, or the involvement of simulation scenarios in situ in the real working environment. These simulations are designed to replicate a longer, complex patient situation from start to finish, involving multiple team members and several clinical skills as opposed to a singular focus, which allows participants to develop technical skills with the additional benefit of continuously emphasising nontechnical skill growth, including communication and problem solving [33]. Subsequently, institutions who incorporate VR into surgical training as standard may find more significant results, including higher checklist scoring, by embedding their VR simulation usage into a complete OR setting, including pre- and post-op steps.

The highest Kirkpatrick level requires analysis of the long-term results of training—something that has yet to be documented in the literature, with current studies focusing on results immediately after training. As the breadth of knowledge about the effectiveness of VR simulation in orthopaedic training increases with the publishing of more RCTs, the question being asked should pivot from ‘is this an acceptable teaching tool?’ to ‘does this lead to more successful surgeons, and as a result, better patient outcomes?’. To provide conclusive justification for the integration of VR into orthopaedic training, and indeed for any medical speciality, future studies should aim to answer this question by measuring the impact on trainees in real surgical environments over a longer period.

The quality of certain studies included within this analysis was also questioned through risk of bias and CASP assessment—the quality of the studies was generally found to be of low risk; however, there were some concerns identified. Inconsistences in post-test assessment by Lohre et al. [15] and Hooper et al. [28] may have affected the strength of the results. During CASP analysis, certain studies were notably lower quality than others; Hou et al. [23] had a concerning level of bias and did not adequately fulfil several CASP criteria, including blinding and equal treatment of participant groups, and as such the results of their study should be interpreted with some caution. Conversely, Cannon et al. [19] was judged to be of a particularly high quality, due to its excellent blinding and randomisation, as well as having a relatively large study population, giving a greater weight to their conclusion. Likewise, Banaszek et al. [18] was deemed to be good quality, particularly due to their use of one single-blinded assessor throughout, reducing the risk of detection and assessor bias and increasing the repeatability of their results and validity of their conclusion.

### The future of virtual reality

Modern and immersive methods of surgical simulation are important in order in develop essential skills and confidence in trainees. In a survey of over 500 orthopaedic trainees, 93% stated that they did not feel comfortable when performing their first arthroscopy, and over half of respondents stated they performed at least 20 arthroscopies before they began to feel comfortable. Of the same group, 74% believed that having a skills lab with a dedicated VR simulator is important for orthopaedic training, while only 20% reported having access to one [34]. VR simulation has been deemed to provide a realistic and enjoyable surgical experience, both anatomically and using instruments, and critically, provides a safe and non-threatening environment where trainees can hone their skills [35].

Despite this, there are a number of challenges that have limited VR’s inclusion in the orthopaedic curriculum thus far including the narrow range of skills that can be developed on any one simulator; whilst newer simulators have become more of a multi-tool platform that are able to switch from knee to shoulder to hip, these are still limited to a single procedure, i.e arthroscopy or pedicle screwing. Simulated tasks outside of these are yet to be incorporated, for example ligament reconstruction, and as such, institutions may feel that simulators are not yet cost effective, with individual simulators costing up to 6-figure sums. Therefore, the development of a comprehensive VR-based simulation skills lab will require a significant initial investment from institutions. However, as VR becomes more popular and moves more into mainstream teaching, it is likely that these costs will decrease, and even with costs as they stand, VR may still provide a more cost-effective training tool than current training, with in-surgery training costs estimated to be in the tens of thousands per year [36]. Additionally, when fully developed orthopaedic VR simulators were initially being explored, there was a lack of validation studies providing sufficient evidence that these simulators were accurately replicating the procedure they were emulating, which may have led to hesitancy from institutions to implement them into teaching. More recently, as VR has become more popular, there is a consistently expanding body of validation studies for individual VR simulators. However, these studies have raised an additional challenge for VR, as whilst statements regarding realism of external appearance, displays and instrumentation use are generally agreed with by participants, the realism of the haptic features of both bone and soft tissue is not reliably viewed as realistic [37, 38], a feature that VR developers should focus on in order to provide a more fulfilling simulation experience.

As previously described, the transferability of skills learnt via VR into actual surgical environments has not been widely researched, with only 2 of the 16 studies included in this study examining skills in the OR. Firmly establishing this transferability should be a key outcome for research moving forward, particularly as the generalisability of skills of trainees learning on VR was directly questioned by Middleton et al.

A ‘Task List’ designed for trainers using VR in surgery was proposed in 2018 that addressed some of the concerns raised by almost all reviews on this topic to date [39]. The 7-point list includes recommendation to identify the skills that can and cannot be developed through simulation, to incentivise long-term use of the VR simulator by trainees, to demonstrate the ‘ultimate goal’ of transferability to OR, and—critically—to recognise that VR are not a total substitute for other methods of simulation, notably cadaveric training. This study is in agreement with this set of goals—the results of this analysis show that it is still not transparent that VR is statistically more effective at teaching skills than current simulation and teaching methods, yet it demonstrates a clear potential for an engaging supplementation to current ways of learning. Future research should aim to address these recurrent topics, in order to help drive the inclusion of VR into surgical curriculum forward.

### Limitations

Although this study performed analysis on 16 articles, the total number of participants was only 431, with an average number of 27. As already previously described, there was a level of heterogenicity across the studies, making comparisons more difficult.

Additionally, the eligibility criteria defined in this study limited available articles to RCTs, due to the level of evidence that they provide, and the ability to make direct comparisons to current educational techniques. However, there are noteworthy limitations to using RCTs in medical education-based research; there are common weaknesses in participant eligibility, methods of randomisation and blinding which can lead to several biases, including performance bias [40]. Indeed, several studies did demonstrate concerns around randomisation and blinding that may affect the quality of their results, and only Sugand et al. [27] actively attempted to reduce participant selection bias by recruiting participants through a mandatory course.

## Conclusion

Virtual reality presents as an immersive new simulation technology that has been adopted by many disciplines, but is underused in the field of orthopaedics. The results of numerous RCTs show it to be proficient in teaching orthopaedic surgical skills, often leading to better participant outcomes compared to existing low-fidelity simulators. However, there are still gaps in the evidence to support VR, crucially that VR learning transfers into the operating room and exploring this should become the focus of studies moving forward.

## Data Availability

All data generated or analysed during this study are included in this published article.

## Abbreviations

VR: Virtual reality
OR: Operating room
RCT: Randomised controlled trial
GRS: Global rating scale
IM: Intramedullary
FAST: Fundamentals of Arthroscopic Surgery Training
GOALS: Global Operative Assessment of Laparoscopic Skills
THA: Total hip arthroscopy
OSATS: Objective Structured Assessment of Technical Skills
ASSET: Arthroscopic Surgery Skill Evaluation Tool
SS: Statistically significant
